# Precipitation characteristic changes due to global warming in a high‐resolution (16 km) ECMWF simulation

**DOI:** 10.1002/qj.3432

**Published:** 2019-01-09

**Authors:** Xuelei Feng, Chuntao Liu, Feiqin Xie, Jian Lu, Long S. Chiu, George Tintera, Baohua Chen

**Affiliations:** ^1^ Center for Climate Physics Institute for Basic Science Busan South Korea; ^2^ Pusan National University Busan South Korea; ^3^ Department of Physical and Environmental Sciences Texas A&M University‐Corpus Christi Corpus Christi Texas; ^4^ Pacific Northwest National Laboratory Richland Washington; ^5^ Department of Atmospheric, Oceanic, and Earth Sciences George Mason University Fairfax Virginia; ^6^ Department of Mathematics and Statistics Texas A&M University‐Corpus Christi Corpus Christi Texas

**Keywords:** climatological zone, climatology, global warming, high‐resolution ECMWF model, precipitation characteristics, probability density function

## Abstract

Changes in precipitation amount, intensity and frequency in response to global warming are examined using global high‐resolution (16 km) climate model simulations based on the European Centre for Medium‐range Weather Forecasts (ECMWF) Integrated Forecast System (IFS) conducted under Project Athena.

Our study shows the increases of zonal‐mean total precipitation in all latitudes except the northern subtropics (15°–30°N) and southern subtropics‐to‐midlatitudes (30°–40°S). The probability distribution function (PDF) changes in different latitudes suggest a higher occurrence of light precipitation (LP; ≤1 mm/day) and heavy precipitation (HP; ≥30 mm/day) at the expense of moderate precipitation reduction (MP; 1–30 mm/day) from Tropics to midlatitudes, but an increase in all categories of precipitation in polar regions.

On the other hand, the PDF change with global warming in different precipitation climatological zones presents another image. For all regions and seasons examined, there is an HP increase at the cost of MP, but LP varies. The reduced MP in richer precipitation zones resides in the PDF peak intensities, which linearly increase with the precipitation climatology zones. In particular in the Tropics (20°S to 20°N), the precipitation PDF has a flatter distribution (i.e. HP and LP increases with MP reduction) except for the Sahara Desert. In the primary precipitation zones in the subtropics (20°–40°) of both hemispheres, precipitation over land switches toward higher intensity (HP increases, but MP and LP decrease) in both winter and summer, while precipitation over ocean in both seasons shows a flattening trend in the intensity distribution. For the major precipitation zones of the mid‐to‐high latitude belt (40°–70°), PDF of precipitation tends to be flatter over ocean in summer, but switches toward higher intensities over land in both summer and winter, as well as over ocean in winter.

## 1 INTRODUCTION

The global mean atmospheric hydrological cycle is projected to intensify with the global warming due to the increased anthropogenic greenhouse gas emission. Global mean precipitation has been found to increase in both observations (Gu *et al*., 2007; Wentz *et al*., 2007) and climate model simulations (Meehl *et al*., 2007; Vecchi and Soden, 2007), though the magnitude of this increase remains uncertain. With global‐mean precipitation constrained by the energy balance between latent heating and radiative cooling (Allen and Ingram, 2002; Trenberth, 2009), climate models predict global‐mean precipitation increases of 1–3% K^−1^ in response to surface warming (Held and Soden, 2006; Sun *et al*., 2007), while observations show the rate of increase with a much wider range among different datasets. For example, Wentz *et al*. (2007) argued that precipitation and total atmospheric water have increased at about the same rate (∼6% K^−1^) as extreme precipitation over the past two decades. Adler *et al*. (2003) used the Global Precipitation Climatology Project (GPCP) precipitation and National Aeronautics and Space Administration (NASA) Goddard Institute for Space Studies (GISS) surface temperature, to estimate a rate of 2.3% K^−1^ increase between 1979 and 2006.

Patterns of precipitation and storm events are likely to change as well since precipitation is directly impacted by changes of atmospheric circulation in addition to the increases in water vapour with a warmer climate. In comparison to global‐scale temperature change, precipitation changes on a regional scale are more complex and show poor agreement among climate models (Knutti and Sedláček, 2012; Shepherd, 2014). However, most models agree that precipitation will increase in equatorial and high‐latitude regions and decrease in subtropical areas, roughly matching the prediction of the “rich‐get‐richer” mechanism (e.g. Chou and Neelin, 2004; Held and Soden, 2006; Boucher *et al*., 2013). In this mechanism, the increased water vapour gives rise to the positive (negative) precipitation anomalies over climatological wet (dry) regions, provided that circulation is unchanged, only carrying more moisture around. Negative precipitation anomalies near the convective margins are associated with the “upped ante” mechanism, in which dry advection transports relatively dry air from areas outside convective regions and suppresses convection over the convective margins (Chou and Neelin, 2004).

“Wet places become wetter and dry places become drier” has been demonstrated with climate model simulations in the past, but the details of the variations of precipitation as a function of intensity over dry and wet regions have yet to be examined in a more systematic manner. In particular, the amount of precipitation is not only controlled by the intensity but also by the frequency. A number of model studies suggest that there is more intensive precipitation but less precipitation frequency resulting from global warming (Wilby and Wigley, 2002). In a warmer climate, moisture content available for extreme events tends to increase at a rate of ∼7% K^−1^ with constant relative humidity, which is governed by the Clausius–Clapeyron equation. Heavy Precipitation intensity is expected to increase at approximately the same rate because the precipitation rate during a storm is proportional to the low‐level moisture convergence (Trenberth *et al*., 2003), while the increase in global mean precipitation is merely 1–3% K^−1^ due to the energy constraints. The discrepancy between heavy precipitation and mean precipitation changes implies that precipitation frequency should decrease in order to offset the large increase in extreme precipitation (Trenberth *et al*., 2003). Later, observations and model simulations obtained consistent results that extreme precipitation tends to become more frequent in most regions (Groisman *et al*., 2005) and the light to moderate precipitation becomes less frequent (Fujibe *et al*., 2005; Sun *et al*., 2007). However, the increasing rate of precipitation extremes simulated with climate models does not match the rate of water vapour content changes (O'Gorman and Schneider, 2009; Lu *et al*., 2014).

Although changes in precipitation characteristics have been investigated by many previous studies, conclusions remain substantially diverse on the precipitation changes at regional scale even for relatively large spatial averages (Allan and Soden, 2007; 2008; Chou *et al*., 2007; Trenberth and Dai, 2007). The preceding analyses usually simply divided precipitation into light, moderate and heavy categories using very limited intervals. This study, however, utilizes dense intensity intervals to systematically examine the probability distribution function (PDF) of changes in both frequency and amount. The regional variations of precipitation characteristic changes are discussed by categorizing the areas into different latitudes and different precipitation climatological zones.

## 2 DATA AND MODEL CONFIGURATION

This study is based on the atmospheric general circulation model (AGCM) simulations from Project Athena. Project Athena is an international collaboration involving five institutions, in response to the call for a revolution in seamless weather and climate modelling made at the World Modelling Summit, held in May 2008 in Reading, United Kingdom (Shukla *et al*., 2009). It brought together an international team including climate and weather scientists and modellers, as well as experts in high‐end computing (HEC). The Athena supercomputer, a dedicated computing resource, operated by the University of Tennessee's National Institute for Computational Science (NICS) and hosted by Oak Ridge National Laboratory (ORNL), is used to run the experiments. Numerical simulations were carried out with two different global atmospheric models: European Centre for Medium‐range Weather Forecasts (ECMWF) Integrated Forecast System (IFS) model, and the Non‐hydrostatic Icosahedral Atmospheric Model (NICAM) from the Japan Agency for Marine–Earth Science and Technology (JAMSTEC) and the University of Tokyo. Kinter *et al*. (2013) provide an excellent summary of Project Athena.

Multiple numerical simulations were conducted for this project, but only Atmospheric Model Intercomparison Project (AMIP) style and Time‐slice experiments with IFS are presented in this article. The IFS solves the hydrostatic primitive equations using two‐time‐level, semi‐implicit semi‐Lagrangian discretization (Ritchie *et al*., 1995; Temperton *et al*., 2001). The spectral space is applied to compute horizontal derivatives and the Helmholtz problem associated with the semi‐implicit time stepping. The spectral transform method is used for the computation of advection, the physical parametrizations, as well as the nonlinear terms so that they can be conducted on the linear reduced Gaussian grid (Hortal and Simmons, 1991). IFS integrations have four different horizontal resolutions, which were named according to the triangular cut‐off wave number in the spherical harmonics expansion of the spectral model: T159 (126 km), T511 (39 km), T1279 (16 km) and T2047 (10 km), corresponding respectively to 320, 1,024, 2,560 and 4,096 grid points along the Equator (Feng *et al*., 2017). IFS is discretized with 91 hybrid vertical levels (top full level at 0.01 hPa) using a finite‐element scheme (Untch and Hortal, 2004). The coupling between the dynamics and physics and the individual parametrization schemes is given by Beljaars *et al*. (2004). The convection parametrization is based on a mass flux scheme described in Tiedtke (1989). Details of the original prognostic cloud scheme are given in Tiedtke (1993). The boundary‐layer scheme is discussed by Beljaars (1995). The radiation package is described in Morcrette *et al*. (2008), and the land surface scheme is explained in Viterbo and Beljaars (2005) and Balsamo *et al*. (2009). Most of the parametrization schemes were left unchanged across all resolutions. For the convection scheme, however, the convective adjustment time is resolution dependent, and is longer in relatively lower resolutions (i.e. going from T159 to T511) (Jung *et al*., 2012). All atmosphere‐only integrations with the IFS were carried out with the prescribed forcing from observed sea‐surface temperature (SST) and sea ice fields. The detailed observation sources and all types of experiments are described in Jung *et al*. (2012).

### 2.1 AMIP‐style integrations

Continuous integrations over the 47‐year period from January 1961 to December 2007 forced by the observed history of SST and sea ice fields, similar to the ones carried out in the Atmospheric Model Intercomparison Project (AMIP: Gates, 1992), were implemented with IFS at T159 and T1279. In the AMIP‐style runs, atmospheric initial conditions and lower boundary fields are a combination of T159 data from the 40‐year ECMWF Re‐Analysis (ERA‐40, covering 1960–1989) and T255 data from ERA‐Interim (1990–2007). These data are monthly before 1990 and weekly starting from 1990, and are then interpolated to daily values and spatially to the grid of the model. For the period 1960–81, SST and sea ice fields used in AMIP runs are produced by the UK Met Office (Rayner *et al*., 2003). The National Oceanic and Atmospheric Administration/National Centers for Environmental Prediction (NOAA/NCEP) two‐dimensional variation data assimilation (2DVAR) dataset (Reynolds *et al*., 2002) was then used from 1981 to 2001. Beginning from 2002, daily SST and sea ice data from the operational ECMWF analysis were adopted.

### 2.2 Time‐slice experiments

The Time‐slice (TS) experiment is run by including the boundary conditions representing the end of twenty‐first century situations under climate change. With the IFS and covering the period 2070–2117, this experiment was also carried out at T159 and T1279. The forecast was started using atmospheric initial conditions of ERA‐40 from 1 November 1970. Both SST and sea ice changes were taken as the difference between 2065–2075 and 1965–1975 monthly climatologies from the Community Climate System Model (CCSM) simulation with the assumption of the atmospheric greenhouse gas and aerosol concentrations following the Intergovernmental Panel on Climate Change (IPCC) emissions scenario A1B until the year 2100, thereafter being constant at their 2100 values (Collins *et al*., 2006). Monthly values were then linearly interpolated to daily values. The atmospheric greenhouse gases of this experiment also follow the A1B scenario (Nakicenovic *et al*., 2000), but the aerosol concentration is the same as the one in the AMIP run. Besides the underlying higher temperature forcing, the higher A1B‐scenario greenhouse gases would result in additional warming of the atmosphere in this experiment.

Only simulations at T1279 resolution for both AMIP and TS experiments are analysed because its resolution is similar to that of the observed dataset chosen for comparison. The difference between TS and AMIP runs will be referred to as the response to climate warming in the context of this study. For comparison purposes, the 6‐hourly precipitation data from simulations and 3‐hourly data from observations are both aggregated into daily data.

To validate the model simulations, the observed research‐grade 3‐hourly precipitation data (3B42) from the Tropical Rainfall Measuring Mission (TRMM) Multisatellite Precipitation Analysis (TMPA) products are used. The TMPA dataset is reprocessed using the Global Precipitation Measurement (GPM) era algorithms to generate a continuous record with the Integrated Multisatellite Retrievals for GPM (IMERG) multisatellite dataset. This dataset has a horizontal resolution of 0.25° × 0.25° (Huffman *et al*., 2010). It spatially covers from 50°S to 50°N, and temporally it can trace back to 1998 (Feng *et al*., 2017). We adopt the 3B42 precipitation data from 1998 to 2017 in this study.

## 3 CHANGES OF PRECIPITATION RATES UNDER A WARMER CLIMATE

### 3.1 Changes of precipitation amount, intensity and frequency in different latitudes

#### 3.1.1 Zonal mean distribution of the precipitation changes

**Figure 1:**
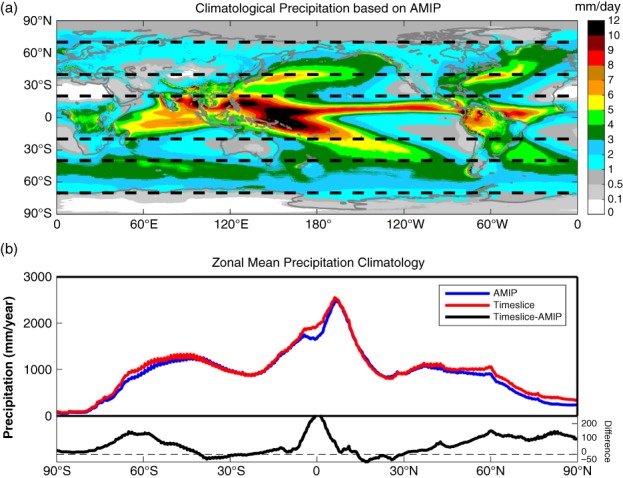
(a) Geographical distribution of daily precipitation climatology from AMIP simulation. (b) (top) Latitudinal distributions of zonal‐mean precipitation climatology from AMIP (blue line) and Time‐slice (TS; red line); (bottom) zonal‐mean climatological precipitation difference (TS−AMIP; black line) indicating the precipitation change under global warming [Colour figure can be viewed at wileyonlinelibrary.com].

Figure 1a illustrates the precipitation climatology simulated by IFS AMIP simulation. This geographical distribution captures the well‐known features of the observed global distribution of total precipitation. The top panel in Figure 1b represents the latitudinal distribution of zonal‐mean precipitation for both AMIP and TS. The lower panel in Figure 1b is the difference between the TS and AMIP runs, which demonstrates the changes of zonal‐mean precipitation associated with global warming. The meridional structure of the changes is in general consistent with previous simulations (Sun *et al*., 2007) (i.e. large increases tend to occur in the rainy Tropics with a pronounced peak around the Equator; large decreases occur in dry subtropics; positive anomalies in mid–high latitudes occur on both hemispheres). However, the peaks of the response do not coincide with the local maxima in the climatological zonal‐mean precipitation; instead, the midlatitude peaks tend to shift poleward in both hemispheres. These shifts suggest that climate warming influences not only the water‐holding capacity, but also the larger‐scale circulations (Lorenz and DeWeaver, 2007; Lu *et al*., 2007; Chadwick *et al*., 2013; Huang, 2014). Chou *et al*. (2009) revealed the reduction of tropical circulation can be largely attributed to the combination of the upped‐ante mechanism and the convection‐deepening on a regional basis. According to the zonal‐mean precipitation distribution (Figure 1b), we will examine the change patterns separately in section 3.2 for the Tropics (20°S to 20°N), subtropics (20°–40°), and mid‐to‐high latitudes (40°–70°) as marked in Figure 1a by dashed lines.

Measuring changes in precipitation frequency and intensity is also crucial to assessing the impacts of global warming (Trenberth *et al*., 2003). To further understand the detailed precipitation changes under a climate‐warming scenario, the PDFs of precipitation frequency and accumulation in different latitudes are investigated. For a given latitude, the PDF is obtained by assembling all daily precipitation data of that latitude, and computing the histogram based on these data. This histogram is then normalized by the number of years. The binning for the histogram calculation is set, according to the precipitation intensity, into three segments including 90 elements in total. The first segment has five bins: 0–0.05, 0.05–0.1, 0.1–0.2, 0.2–0.3 and 0.3–0.4 mm/day; it is followed by18 evenly distributed bins between 0.6 and 4.0 mm/day with the interval of 0.2, plus 67 additional exponential bins from exp[log(5000)/100*17] up to 1,000 mm/day. The design of the sampling bins allows us to construct smooth PDFs spectra in different latitudes, thus it is applied to all latitudes. For the convenience of discussion, the precipitation intensity is divided into three major categories: heavy precipitation (HP; ≥30 mm/day corresponding to ≥1.5 in log_10_‐scale), moderate precipitation (MP; 1–30 mm/day corresponding to 0–1.5 in log_10_‐scale), and light precipitation (LP; ≤1 mm/day corresponding to ≤0 in log_10_‐scale).

**Figure 2:**
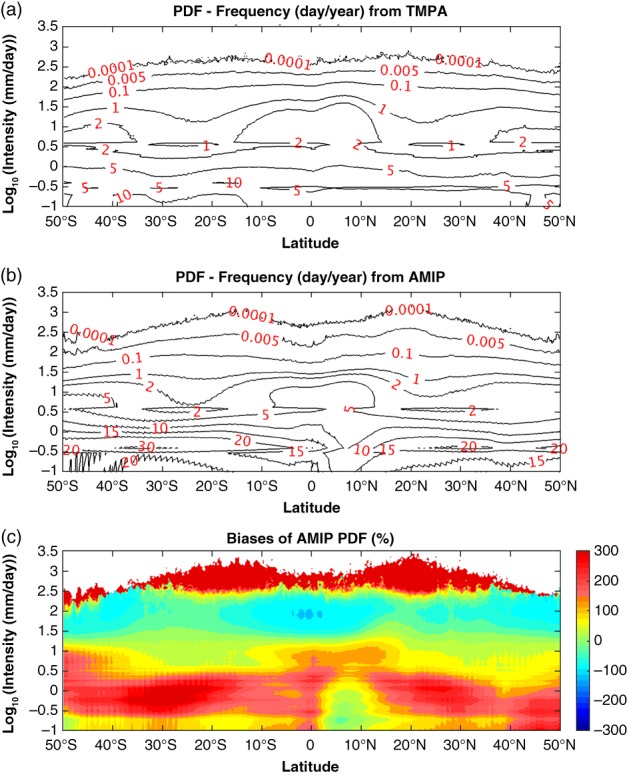
The PDFs of precipitation frequency in different latitudes (50°S to 50°N) from (a) TMPA, (b) AMIP simulation, and (c) the fractional biases (AMIP−TMPA)/TMPA*100. Intensities (*y*‐axis) are set in the log_10_‐scale. The PDF of frequency in each intensity bin is the average number of days per year when the precipitation rate falls within the bin. In each latitude, the summation of frequency PDF across precipitation intensity spectrum in (a) and (b) is 365 days [Colour figure can be viewed at wileyonlinelibrary.com].

Before exploring the global warming influences on the PDF change, we first evaluate the PDFs from the AMIP run in different latitudes against the TMPA observations (Figure 2). The spectral pattern from the AMIP run (Figure 2b) shows notable resemblance to that of TMPA (Figure 2a). They both have the general tendency of decreasing frequency with the intensity at all latitudes. The extreme precipitation has a relative minimum at the Equator, increasing to a maximum at ∼15° in both hemispheres, before tapering to their absolute minimum at the far ends of latitude. In both panels, there are three spectral centres in the intensity of ∼10 mm/day (corresponding to 1 in log_10_ scale). The major centre resides at the Tropics corresponding to the intertropical convergence zone (ITCZ), and the other two at the midlatitudes corresponding to the storm tracks of both hemispheres. On the other hand, the bias panel (Figure 2c) presents an overestimate of LP and MP, but an underestimate of the HP, which is common to current climate models (e.g. Dai, 2006). Moreover, we need to point out that the extremely heavy precipitation (≥300 mm/day; ∼2.5 in log10 scale) is overestimated in AMIP simulation.

**Figure 3:**
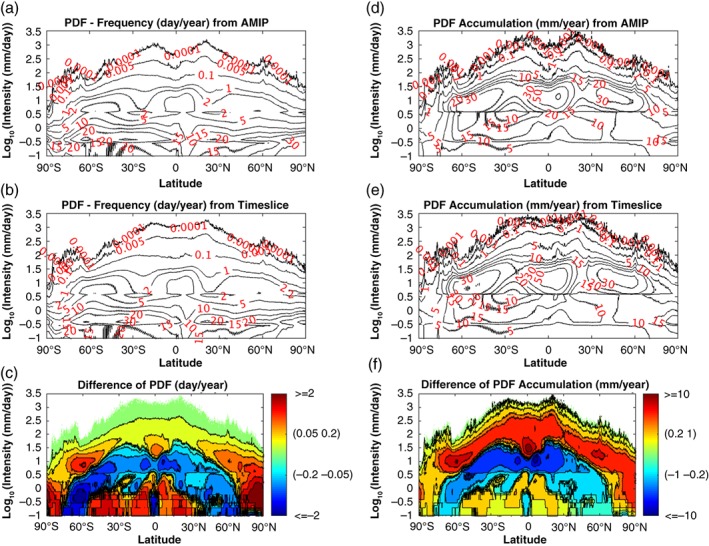
The PDFs of precipitation frequency in different latitudes (90°S to 90°N) from (a) AMIP, (b) TS, and (c) their difference (TS−AMIP). The PDFs of precipitation accumulation in different latitudes from (d) AMIP, (e) TS, and (f) their difference (TS−AMIP). Intensities (*y*‐axis) are set in the log_10_‐scale. The PDF of frequency in each intensity bin is the average number of days per year when the precipitation rate falls within the bin. Similarly, the PDF of precipitation accumulation sums up the annual accumulation of precipitation for each intensity bin. In each latitude, the summation of frequency PDF across precipitation intensity spectrum in (a) and (b) is 365 days, and the summation of the PDF accumulation across precipitation intensity spectrum in (d) and (e) is the total annual mean precipitation amount, respectively [Colour figure can be viewed at wileyonlinelibrary.com].

We now examine the changes between AMIP and TS experiments in terms of both the PDF of frequency and PDF of precipitation accumulation. The total PDFs in Figure 3a,b illustrate that most of the precipitation falls as LP in both AMIP and TS runs. However, the MP contributes most to the total precipitation, especially in the Tropics and the mid–high latitudes (Figure 3d,e). Figure 3c,f show the PDF changes of precipitation frequency and accumulation. These two panels show consistent patterns by definition. The differences between Figure 3c and Figure 3f lie in the relative magnitude. The overall change of the precipitation PDFs in different latitudes (Figure 3c,f) shows considerable resemblance to the global warming induced PDF change in Lu *et al*. (2014) using Community Atmosphere Model Version 3.0 under an aquaplanet configuration, as well as the precipitation PDF change in Coupled Model Intercomparison Project Phase 3 (CMIP3) models (Pall *et al*., 2007). The most remarkable feature is the increase in HP frequency over all latitudes, which has been noticed by several observational and modelling studies (Houghton *et al*., 2001; Emori and Brown, 2005; Held and Soden, 2006; Zhang *et al*., 2007). It suggests that heavy precipitation will happen more frequently in the future under a warmer scenario. The frequency change of light precipitation is stronger than that of the heavy precipitation (Figure 3c). But this situation is just reversed in the precipitation accumulation panel (Figure 3f). In general, between 40°N and 60°S excluding the equatorial region, precipitation PDFs tend to increase in LP and HP but decrease in MP, while within the latitudinal bands of 40°–70°N and 60°–80°S LP, decreases but MP and HP increase in frequency. Poleward of 70°, precipitation of all intensities increases. In addition, compared to other regions in the low latitudes, the narrow equatorial belt stands out with a significant decrease of LP and increase in HP.

#### 3.1.2 Geographic distributions of precipitation changes in different categories and seasons

**Figure 4:**
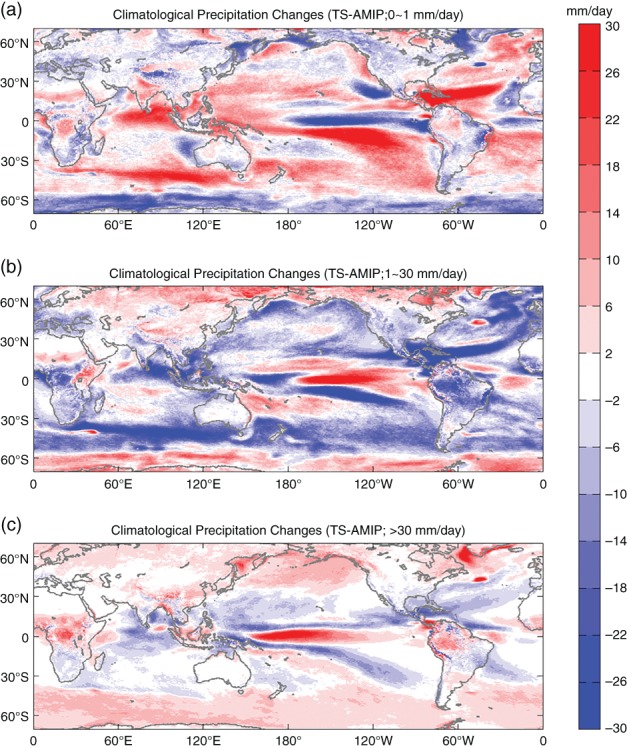
Annual mean precipitation changes (TS−AMIP) for (a) light precipitation (LP) with rate less than 1 mm/day, (b) moderate precipitation (MP) with rate of 1–30 mm/day, and (c) heavy precipitation (HP) with rate larger than 30 mm/day [Colour figure can be viewed at wileyonlinelibrary.com].

Figure 4 shows the geographic distribution of the precipitation response for the three intensity categories defined above. Regions poleward of 70° are not presented because they show a consistent increase in all intensities (Figure 3c,f). Overall, in the Tropics and subtropics wherever there is a decrease of LP, there is an increase of MP and HP, indicating that total increase of precipitation occurs as the result of a spectral shift from weak events to intensive events, and vice versa for the decrease of total precipitation. The most notable is the intensification in the eastern equatorial Pacific sandwiched by the surrounding reduction in the changes of MP and HP. There is an intriguing exception over tropical Africa, where all three categories increase. In the midlatitude band (especially Southern Hemisphere), there is a pattern of increase in LP, decrease in MP, and increase in HP again. After inspecting the PDF distribution of the background accumulative precipitation, this appears to be attributable to the poleward shift of the PDF spectrum near the midlatitudes.

In Figure 3c,f, we also notice an asymmetric precipitation change over mid–high latitudes (40°–60°) between the two hemispheres, i.e. LP increases in Southern Hemisphere (SH) while it decreases in Northern Hemisphere (NH). It is clear that this asymmetry can be attributed to the difference of surface conditions. The ocean‐dominated SH mid‐to‐high latitudes show an increase in LP with global warming, while the continent‐dominated NH midlatitudes exhibit a decrease in LP (Figure 4a). Moreover, the decrease of LP at the Equator in Figure 3c,f results from the southern edge of the central and eastern Pacific ITCZ, and in the equatorial Atlantic (Figure 4a).

**Figure 5:**
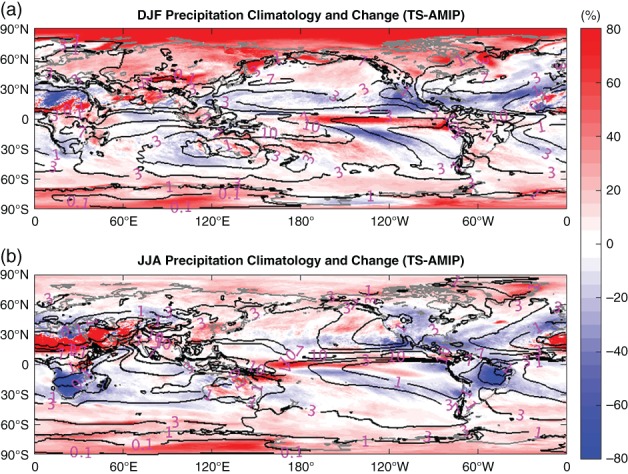
Global distribution of precipitation climatology (contours) simulated by AMIP and the fractional change (shadings; (TS−AMIP)/AMIP*100) under global warming in (a) DJF and (b) JJA [Colour figure can be viewed at wileyonlinelibrary.com].

Climate change has altered not only the overall magnitude of rainfall but also its seasonal distribution (Easterling *et al*., 2000). The geographical distribution of precipitation climatology from AMIP (contour) and their changes (shaded; TS−AMIP) under global warming for December–January–February (DJF) and June–July–August (JJA) are displayed in Figure 5. At latitudes poleward of 70°, precipitation is enhanced in all regions with larger increases in DJF (Figure 5a) than in JJA (Figure 5b). In mid–high latitudes (40°–70°), precipitation increases in most regions in both seasons except in the central United States, where it exhibits a remarkable decline of precipitation in JJA. At midlatitudes, precipitation increases more in DJF than in JJA, especially over land. The impact of land–ocean contrast is also displayed in Figure 4b, which displays MP increases over most of the land areas but decreases over oceans in both hemispheres. The opposite is true for LP (Figure 4a). In the Tropics and subtropics, precipitation change pattern is more regional. The often‐cited conclusion that “wet get wetter and dry gets drier” (Chou and Neelin, 2004; Held and Soden, 2006) was not borne out here. The regions outlined by the 7 mm/day contour in the tropical Pacific roughly correspond to the main rain band of the ITCZ, where precipitation does not change significantly (Figure 5a,b). However, at their southern edges, large positive anomalies are noticed with a sharply increasing rate at ∼60–80%. In addition, Figure 4 shows the opposite phase of precipitation changes in all panels between Pacific ITCZ and its southern edge. This north–south dipole structure of precipitation changes in Figure 4, as well as the position displacement between deep tropical large precipitation increases and ITCZ in Figure 5, indicates that the Indo‐Pacific ITCZ shifts southward. The ITCZ southward shift under warmer scenarios has been documented (Rotstayn *et al*., 2000; Williams *et al*., 2001; Rotstayn and Lohman, 2002). This shift occurs in both DJF and JJA. In DJF, it occurs mostly over the eastern Pacific but it is centred over the central Pacific in JJA. However, the ITCZ in the Atlantic and Africa shifts northward, as indicated by the large increase flanking the northern edge of the ITCZ from the Atlantic coast of Mauritania and Senegal through to Sudan, Eritrea and the Red Sea and Arabian Peninsula, especially in JJA (Figure 5b). Precipitation in the southern part of these regions increases in all categories, as shown in Figure 4. This supports the finding based on model simulations by Dong and Sutton (2015) that rain comes to the Sahel semi‐arid regions in the summer months, as the tropical rain belt moves north from the Equator, and agrees with the conclusion from observations that the southern border of the Sahara shrinks (Mueller, 2011). In addition, there are two land regions (dry Brazilian region, Southern Africa) undergoing a large decrease in precipitation (Figure 5). The decline in Southern Africa and the dry Brazilian region is more significant in austral winter than austral summer. This large precipitation decrease in Southern Africa has been documented in Solomon *et al*. (2007) under the A1B scenario, and the drying in Brazil by Malhi *et al*. (2008) based on 23 climate models.

### 3.2 Change of precipitation rates in different climatological zones

Precipitation systems are significantly different between the convection‐dominated Tropics and baroclinic system‐dominated mid‐ and high latitudes. According to the zonal mean precipitation distribution (Figure 1b), we separately explore the precipitation characteristic responses to global warming in three different latitudinal belts, shown in Figure 1a by dashed black lines, including the Tropics (20°S to 20°N), subtropics (20°–40°), and mid‐to‐high latitudes (40°–70°). Each belt, in terms of the AMIP simulated precipitation climatology, is in turn categorized into 16 precipitation climatological zones using the precipitation rate intervals: 0.1 mm/day and less, 0.5, 1, 2, 3, 4, 5, 6, 7, 8, 9, 10, 12, 14 and 16 mm/day and above. A precipitation climatological zone, denoted as *R*_clim_, is defined according to the precipitation interval that the regional climatological precipitation falls in. For a given *R*_clim_, the corresponding PDF can be constructed by pooling all daily precipitation samples using the same precipitation intensity bins as used in Figure 2. The PDF in each *R*_clim_ is then normalized by the sample size from that *R*_clim_. The summation across precipitation intensity spectrum in any *R*_clim_ is, therefore, 100 (%) for precipitation frequency PDF, and is the daily precipitation climatology for PDF of precipitation accumulation, respectively.

**Figure 6:**
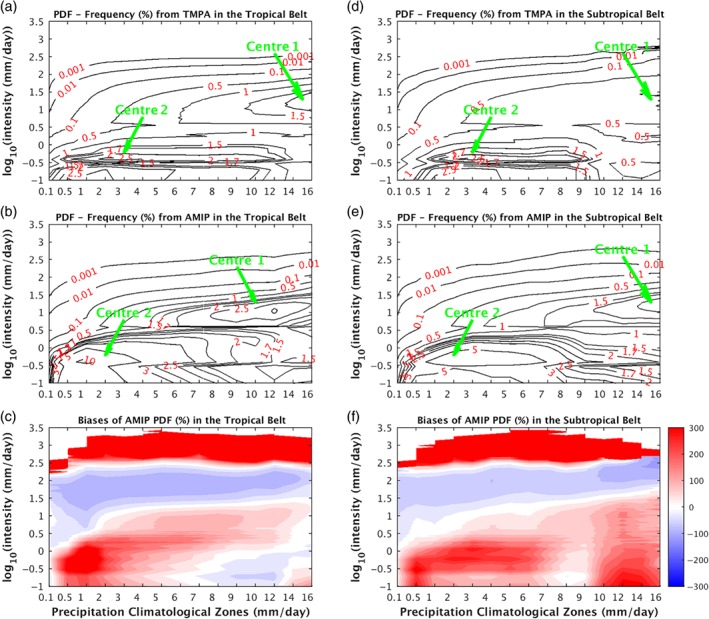
Precipitation frequency PDFs (%) in different precipitation climatological regions (*R*_clim_s). PDFs for the tropical belt (20°S to 20°N; see the dashed black lines in Figure 1a) from (a) TMPA, (b) AMIP, and (c) their fractional biases (shadings) (AMIP−TMPA)/TMPA*100. PDFs for the subtropical belt (20°–40°; see the dashed black lines in Figure 1a) from (d) TMPA, (e) AMIP, and (f) their fractional biases (shadings) [Colour figure can be viewed at wileyonlinelibrary.com].

Prior to examining the precipitation PDF response to the global warming in different climatological zones, we also firstly validate the frequency PDF from the AMIP run against the TMPA observations (Figure 6). Since TMPA precipitation data only cover 50°S to 50°N, the validation of frequency PDF in different climatological zones is only carried out in the tropical belt and the subtropical belt. In the tropical belt, the TMPA PDF panel (Figure 6a) and the AMIP PDF panel (Figure 6b) exhibit similar spectral patterns. They both share the feature of two structure centres: a precipitation‐rich (*R*_clim_ >= 6 mm/day) centre (Centre 1 marked in Figure 6a,b) and a precipitation‐poor (*R*_clim_ <= 4 mm/day) centre (Centre 2 in Figure 6a,b). In addition, the extreme precipitation in both panels increases with the climate zone. The similarity between TMPA and AMIP spectral patterns again suggests some model fidelity in simulating precipitation. Meantime, the biases of AMIP spectral pattern are also readily discernible. The AMIP spectrum overestimates LP and the most extreme HP, but underestimates HP (Figure 6c). The overestimate of LP is a common caveat of climate models, which tend to drizzle too much. As the consequence, since the total precipitation is constrained by the radiative properties of the atmosphere, and hence cannot deviate too much from reality, the simulated HP has to compensate for the overestimate of the LP. The overestimate of extreme HP by the IFS model is curious and no explanation is readily available.

For the climate zones in the Tropics, the modelled Centre 1 has too strong an intensity and resides in poorer precipitation climate zones compared to the TMPA observations (Figure 6c). In the subtropics, the most notable disagreement is that where there is a low Centre 1 in the observation there is now a high Centre 1 in the model simulation (Figure 6f). This might represent a major deficiency of the IFS model in capturing the subtropical precipitation distribution.

#### 3.2.1 Precipitation characteristic changes in the tropical belt

**Figure 7:**
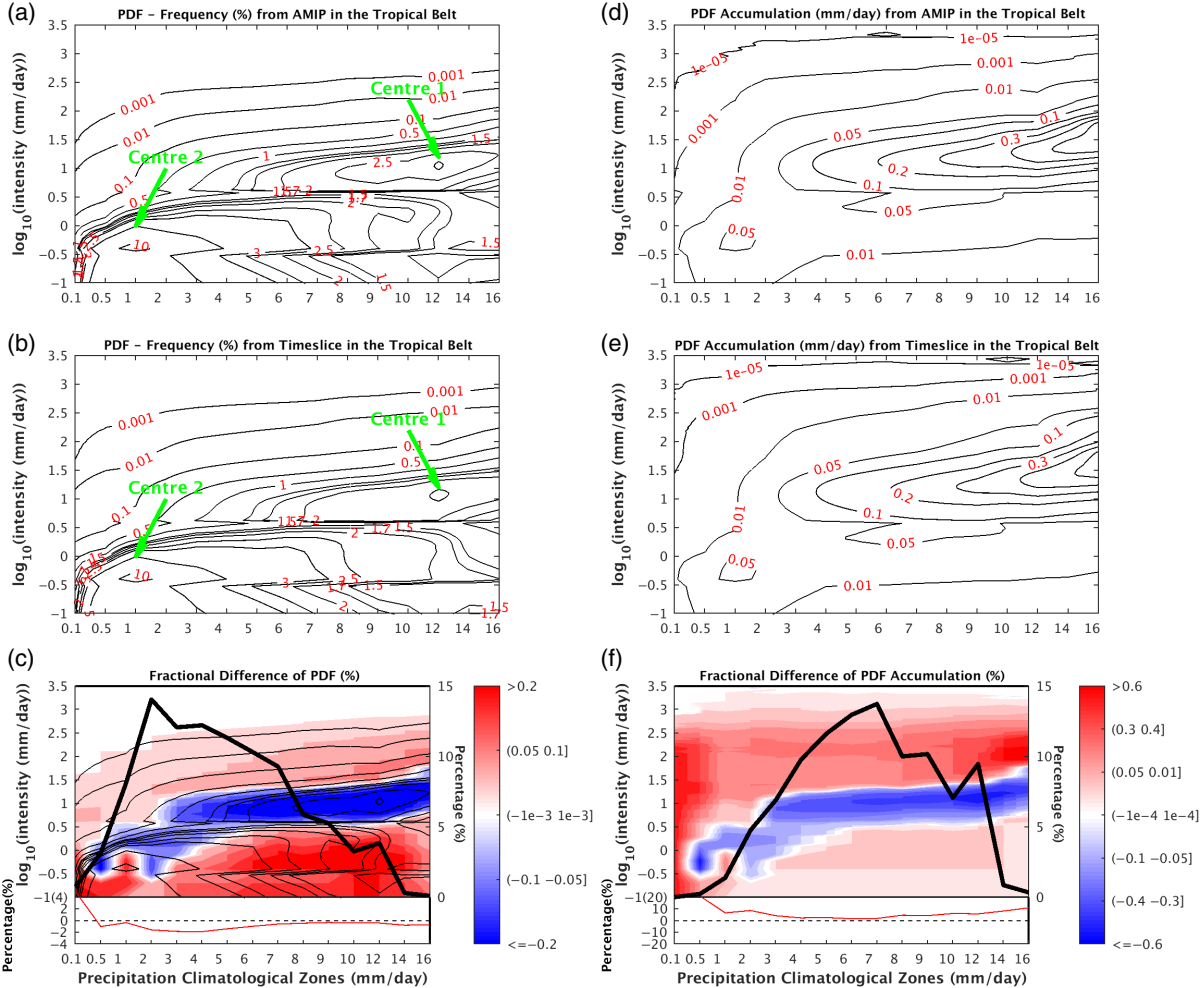
Precipitation frequency PDFs (%) in different precipitation climatological regions (*R*_clim_s) for the tropical belt (20°S to 20°N; see the dashed black lines in Figure 1a) from (a) AMIP, (b) TS, and (c) their fractional difference (shadings) (TS−AMIP)/AMIP*100. Panels (d)–(f) display the PDFs of precipitation accumulation from AMIP, TS and their fractional differences (shadings), in order. The frequency PDF contours from (a) are superimposed in (c). The thick black line in (c) shows the occurrence fractional distribution with *R*_clim_s (i.e. *S*_clim_AMIP_/*S*_tot_*100) from AMIP. Its magnitude is marked in the right‐side *y*‐axis. The red line in the lower part of (c) shows fractional occurrence changes (i.e. *S*_clim_TS−_ *S*_clim_AMIP_/*S*_clim_AMIP_*100) in each *R*_clim_. The thick black line in (f) represents the fractional precipitation amount distribution with *R*_clim_s (i.e. *P*_clim_AMIP_/*P*_tot_*100) from AMIP. The magnitude is likewise marked in the right‐side *y*‐axis, and the corresponding warmer climate‐induced amount changes (i.e. *P*_clim_TS*−*_ *P*_clim_AMIP_/*P*_clim_AMIP_*100) are also shown by red line in the lower part of (f). *S*_tot_ (*P*_tot_) is the total precipitation sample‐size (amount) of the tropical belt from AMIP. *S*_clim_AMIP_ (*P*_clim_AMIP_) represents the precipitation sample‐size (amount) in an individual *R*_clim_ from AMIP, and *S*_clim_TS_ (*P*_clim_TS_) represents the sample‐size (amount) from TS [Colour figure can be viewed at wileyonlinelibrary.com].

Now we study the tropical belt (20°S to 20°N) precipitation PDF changes in different precipitation climate zones. The tropical PDFs of precipitation frequency and accumulation for AMIP and TS, as well as their difference, are displayed in Figure 7. The *x*‐axis in this figure represents the different precipitation climatology zones (*R*_clim_), while the *y*‐direction shows the precipitation PDF for each zone in log_10_‐scale.

The frequency PDFs demonstrate two spectrum centres in both AMIP and TS experiments: Centre 1 (Figure 7a,b) clustering around the peak (12, 0.8) in this *R*_clim_‐intensity diagram, and Centre 2 (Figure 7a,b) clustering around the peak (1, −0.4). The dependence of the log‐scale intensity with climate zones is also revealed. Centre 1 has an ellipse‐like structure with the right side inclining upward. The upward tilt of Centre 1 is, therefore, an indication that the precipitation intensities where the PDF peak lies increase with the rank of precipitation climate zones. Actually, the intensities of the PDF peaks roughly linearly increase with the precipitation climatology when we plot the figure with equal intervals for both axes (not shown). Otherwise said, regions with richer precipitation have heavier main precipitation systems. On the other hand, the modal value of Centre 2 does not vary much with the rank of the climate zones, implying a weak precipitation circulation regime of the atmosphere irrespective of the geographic locations. These two centres are also discernible from the precipitation accumulation PDF (Figure 7d,e), but less clear in poorer precipitation zones.

**Figure 8:**
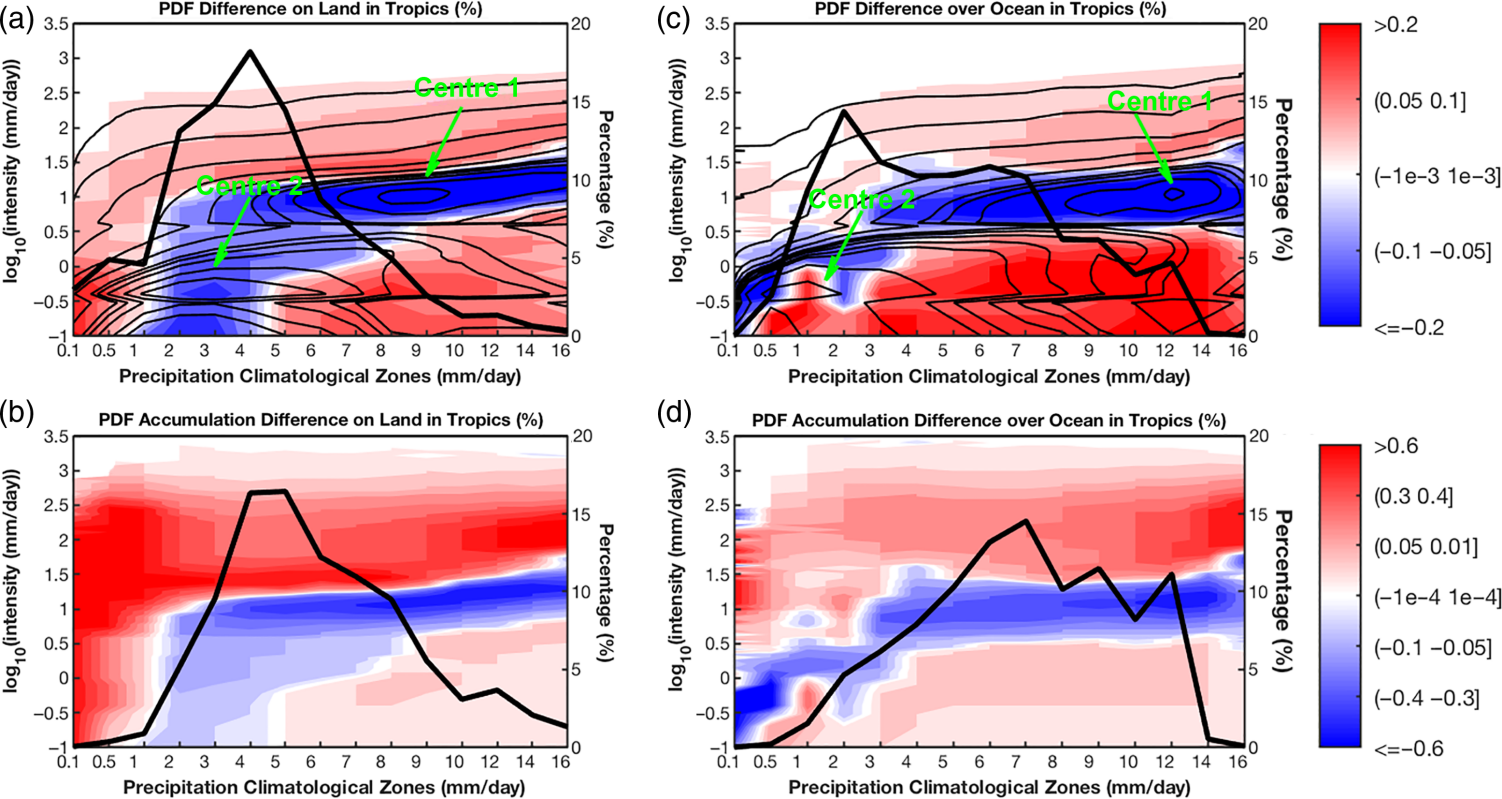
Similar to Figure 7c, upper two panels are the precipitation fractional frequency PDF changes ((TS−AMIP)/AMIP*100) in different precipitation climatological regions: (a) tropical land and (c) tropical ocean. Bottom two panels, similar to Figure 7f, are the fractional PDF changes of precipitation accumulation in different climatological regions: (b) tropical land and (d) tropical ocean. The corresponding precipitation frequency PDFs and the fractional distributions with precipitation climatological regions from AMIP, in both (a) and (c), are also respectively superimposed by contours and thick black lines [Colour figure can be viewed at wileyonlinelibrary.com].

**Figure 9:**
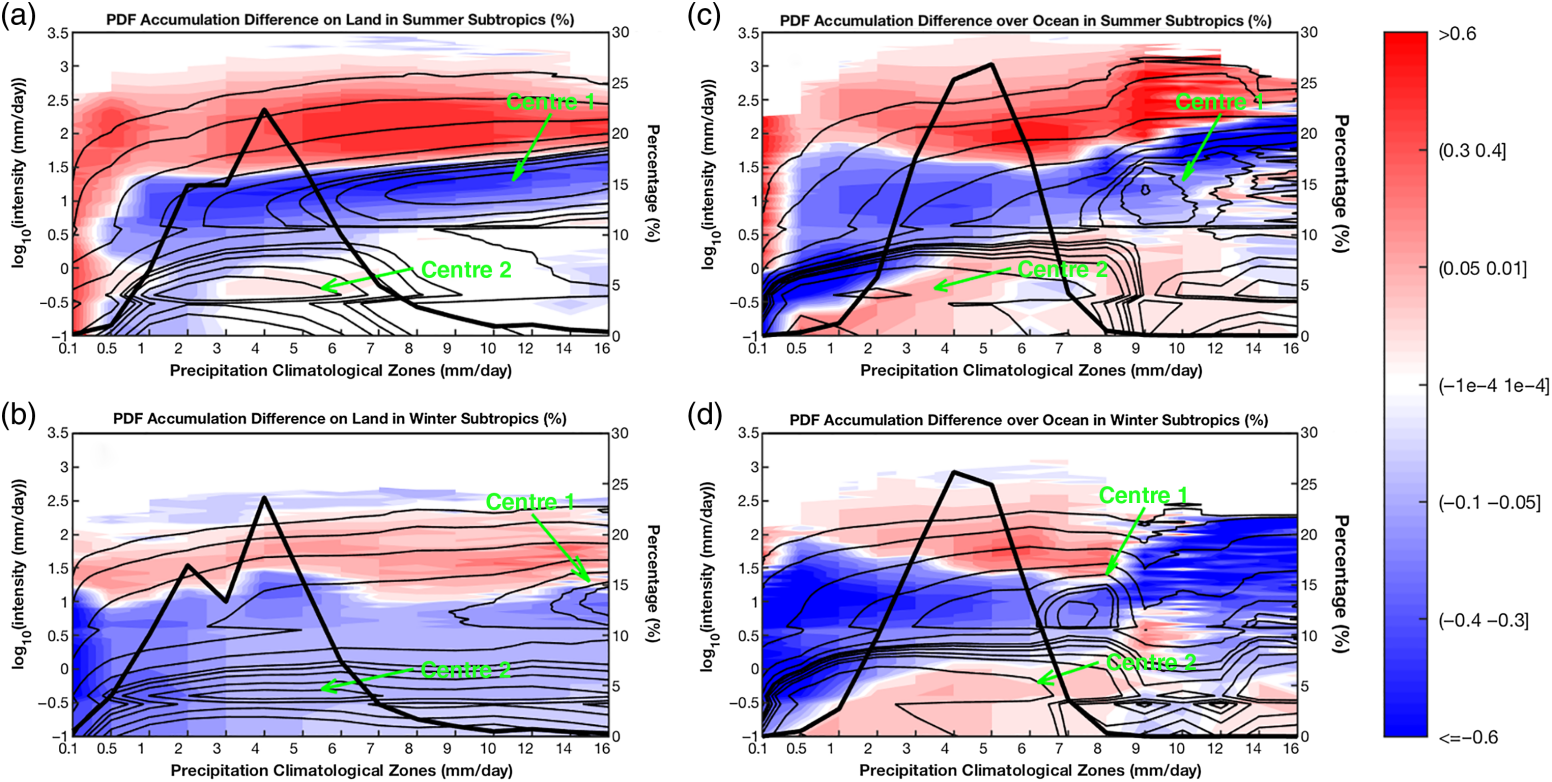
Fractional PDF changes [(TS−AMIP)/AMIP*100] of the precipitation accumulation in different subtropical climatological regions. Each panel is set the same as Figure 7f, but for the subtropics (20°–40°). (a) Summer‐land, (b) winter‐land, (c) summer‐ocean, and (d) winter‐ocean. The frequency PDFs in different climatological regions from AMIP are also overlaid on the corresponding panels by contours [Colour figure can be viewed at wileyonlinelibrary.com].

**Figure 10:**
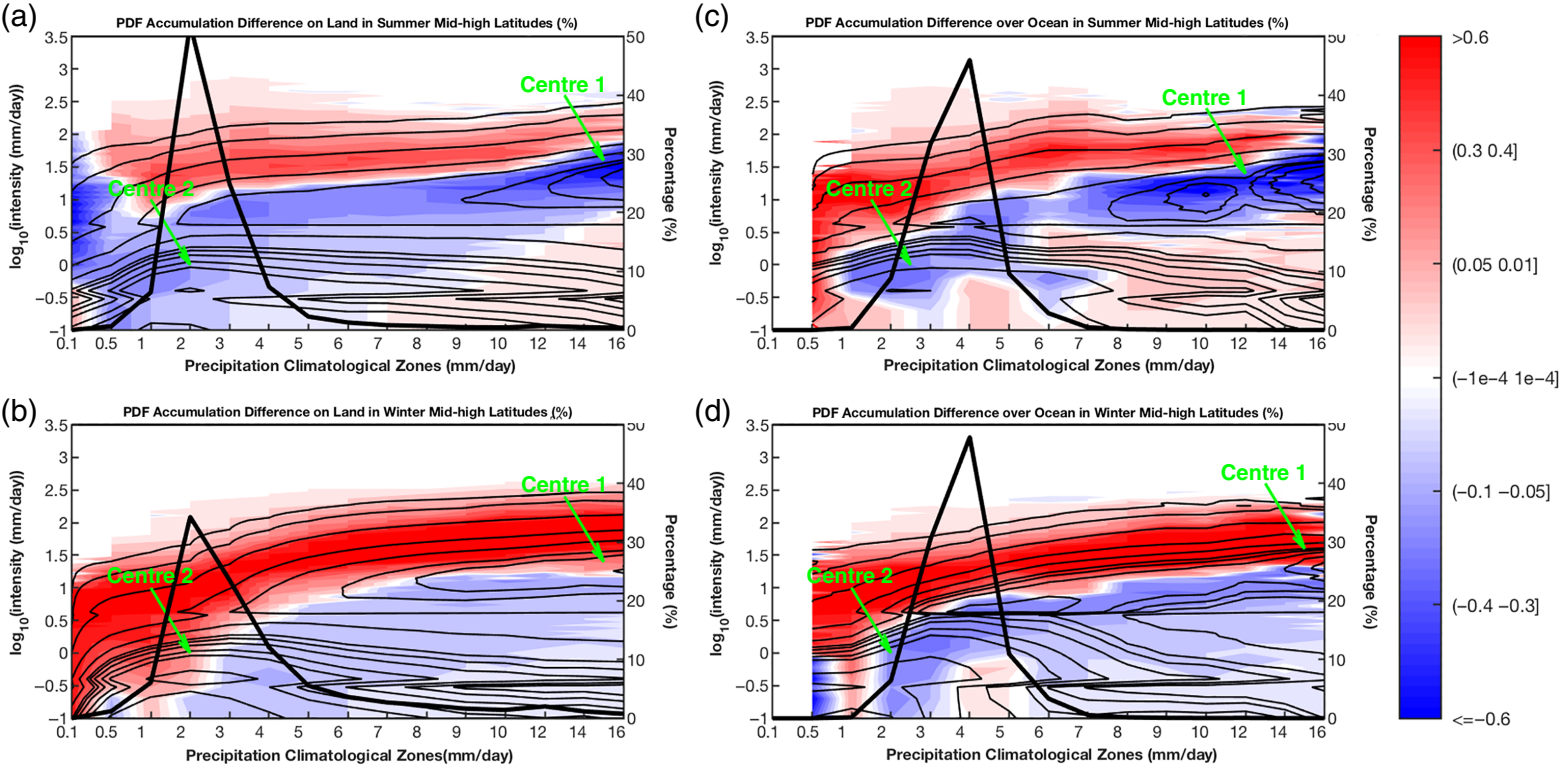
Fractional PDF changes [(TS−AMIP)/AMIP*100] of the precipitation accumulation in different climatological regions of mid–high latitudes (40°–70°). Panels are set the same as Figure 9, but for mid–high latitudes [Colour figure can be viewed at wileyonlinelibrary.com].

Precipitation in the Tropics primarily occurs in 0–14 mm/day precipitation zones with the highest frequency of 2 mm/day, and the most precipitation amounts in the 7 mm/day area (see the thick black lines of Figure 7c,f). Except in the very dry zones (*R*_clim_ < 0.5 mm/day), precipitation becomes less frequent (red line in Figure 7c), but its amount increases in all climatological zones (red line in Figure 7f). The PDF changes of precipitation frequency and accumulation (shadings in Figure 7c,f) both show a band with reduced precipitation (blue colours), sandwiched by enhancements of lighter and heavier types of precipitation (red colours), indicating HP and LP frequencies increase at the expense of MP frequency. We refer to this pattern of PDF spectral change as “flatter distribution.” The overall reduction of the MP frequency regardless of the precipitation climate zones suggests that the tropical ascent in the Centre 1 regime is getting weaker as climate warms. Physical rationale does exist to account for the weakening of the tropical overturning in terms of the constraint on the work output of the atmospheric heat engine (Laliberté *et al*., 2015). The circulation weakening seems to be quite ubiquitous in both Tropics and extratropics and over both land and ocean (Figures 7, 8, 9, 10).

In very dry zones with climatological precipitation rate less than 0.5 mm/day, precipitation of all intensities becomes more frequent and the total precipitation increases. These precipitation climatological zones are mostly in the Sahel region of Africa stretching from the Atlantic coast of Mauritania and Senegal through to Sudan, Eritrea and the Red Sea, as can be seen in Figure 4.

Since mechanisms generating precipitation can differ between tropical ocean and land, a similar analysis is carried out over land and ocean separately (Figure 8). The fractional distributions of precipitation climatology in different zones (thick black lines) show broader spread in all four panels, indicating the Tropics have diverse climatological precipitation from very dry precipitation to very wet precipitation. Both the precipitation frequency (Figure 8a) and climatological amount (Figure 8b) in tropical lands reach the most at around 4–5 mm/day zones, while over tropical ocean the highest frequency occurs at the 2 mm/day zone (Figure 8c), and the largest amounts at the 7 mm/day zone (Figure 8d). Two centres present in the frequency spectrum panels of Figure 7 also emerge in both land and ocean frequency panels (contours in Figure 8a,c). As for the change patterns (shading), the general features in Figure 7c,f are extended to Figure 8, especially to the tropical ocean panels (Figure 8c,d) including the positive tendency in HP and LP and negative tendency in MP. Again, the moderate frequency reduction over ocean graphically follows Centre 1 of the frequency spectra (contours in Figure 8c). This overlap between frequency spectrum Centre 1 and the band with precipitation reduction also happens to the tropical land panel (Figure 8a) in the wet precipitation zones (*R*_clim_ > 5 mm/day). That means the PDF change over wet precipitation land is also characterized as the increase of LP and HP but reduction of MP in frequency and amount. These global‐warming induced features can also be seen in Figure 4. In the zones of *R*_clim_ < 1 mm/day, such as arid regions in Africa (Figure 1a), precipitation of all intensities increases (Figure 8a,b) and thus the total precipitation also increases. Between the dry and rainy zones (1 <= *R*_clim_ <= 5 mm/day), the spectra demonstrate a higher intensity switch tendency, i.e. HP increases at the expense of MP and LP reduction. In addition, the HP increase is larger over tropical land than tropical ocean in all climatological zones. The patterns in the frequency change panels (Figure 8a,c) and in the corresponding accumulation change panels (Figure 8b,d) are very similar. The difference between them lies in the relative magnitude of changes. The increases of HP are stronger than LP in the frequency spectrum change, while this is reversed in the accumulation spectrum change.

#### 3.2.2 Precipitation characteristic changes in the extratropical belts

In extratropical regions, precipitation has strong seasonal dependence. Thus, the PDF differences of daily precipitation accumulation in different climatological zones are examined separately for summer and winter seasons. Summer season is defined as JJA for the NH and DJF for the SH, and vice versa for the winter season. The PDFs are constructed by grouping the data from both hemispheres for a given season, i.e. JJA data from NH and DJF data from SH for the summer season. For brevity, only accumulation PDF and the related changes are presented for the extratropical regions.

For the subtropics (20°–40°), the fractional distributions of precipitation accumulation (thick black lines in Figure 9) have narrower ranges, and peak at drier zones than those in the Tropics (thick black lines in Figure 8b,d). Most of the subtropical regions have precipitation climatology less than 8 mm/day. Over subtropical land in both summer (Figure 9a) and winter (Figure 9b) seasons, the zone with *R*_clim_ = 4 mm/day contributes the most to the total precipitation, but the tropical land (Figure 8c) has the most precipitation at a richer precipitation zone of *R*_clim_ = 5 mm/day. Two subtropical ocean panels (Figure 9c,d) likewise have peaks of accumulation fractional distribution lying in a drier zone than in the tropical ocean panel (Figure 8d). Moreover, Centre 1 and Centre 2 of the frequency spectra, similar to tropical panels of Figure 8, both show up in each of four subtropical panels (contours in Figure 9). These two centres of the frequency spectra in the subtropics (Figure 9) are generally weaker in strength compared to the tropical panels.

Except for the dry zones (*R*_clim_ <= 3 mm/day), the precipitation spectrum changes of summer subtropical land (shadings in Figure 9a) exhibit slightly positive/no change in LP, a positive tendency of HP, but a negative tendency of MP. The major differences between subtropical winter and summer precipitation accumulation PDF changes lie in the LP. The winter‐land panel (Figure 9b) shows a reduction in LP, while the summer‐land panel (Figure 9a) mainly displays slight/no enhancement of LP in the richer precipitation zones (*R*_clim_ > = 3 mm/day). On the other hand, the PDF accumulation of the subtropical ocean panels (Figure 9c,d) in the main precipitation climatology zones (1 <= *R*_clim_ <= 8 mm/day) becomes flatter (e.g. HP and LP increase, but MP decreases). The overlaps between Centre 1 spectrum band and the band of reduction (blue shadings) can also be detected in each panel of Figure 9.

Over mid‐to‐high latitudes (40°–70°), the PDF changes of precipitation accumulation over land and ocean, in both summer and winter seasons are demonstrated in Figure 10. The mid–high latitudes mainly consist of the areas with the precipitation climatology less than 6 mm/day (thick black lines). The zone with *R*_clim_ = 2 mm/day contributes the most precipitation over land (Figure 10a,b), while over ocean, the zone of *R*_clim_ = 4 mm/day does the most (Figure 10c,d). Compared to the Tropics and subtropics, the mid–high latitudes have less precipitation and a narrower range of the main precipitation climatology zones. Within the primary precipitation zones (1 <= *R*_clim_ <= 6 mm/day shown by thick black lines), precipitation accumulation PDF changes (shading) are different over ocean and land. Precipitation on land (Figure 10a,b) tends to become heavier, resulting largely from HP increases, but MP and LP decreases. Note for land in wintertime, zones with *R*_clim_ <= 3 mm/day undergo consistent increases of precipitation at all intensities (Figure 10b). The PDF of precipitation accumulation over summer‐ocean (Figure 10c) is inclined to be flatter, and that over winter‐ocean (Figure 10d) simply shifts to higher intensity. Moreover, two spectrum centres both appear in all mid‐to‐high latitude panels, even though Centre 1 in the winter‐ocean panel (Figure 10d) is less eminent. The position correspondence of Centre 1 with the spectrum band of reduction can be seen in mid‐to‐high latitudes summer as well.

#### 3.2.3 Comparison of precipitation characteristic changes in different latitudinal belts

Generally speaking, high‐latitude regions have less precipitation than low‐latitude regions. Two PDF spectrum centres appear in all seasons and over both land and ocean for all three latitudinal belts. The spectrum centre in higher precipitation climate zones becomes less apparent in a higher latitudinal belt. Comparing the precipitation spectrum change patterns in the three latitudinal belts under climate warming, we find that, except for the very dry zones (*R*_clim_ <= 0.1 mm/day), the spectrum changes in all other zones can be categorized as two typical patterns under global warming: shift towards HP at the cost of LP and MP; and a flatter redistribution of precipitation PDFs featuring a reduction in MP but an increase in LP and HP. Both include the components of HP increases and MP decreases. However, the LP changes can be dependent on seasons and underlying surfaces. The LP for tropical land and ocean, subtropical ocean, and mid‐to‐high latitude summer‐ocean exhibits an increase, while the LP for subtropical and mid–high latitude land, and the mid–high latitude winter‐ocean a reduction.

The revelation of more frequent heavy precipitation is consistent with previous studies (Trenberth *et al*., 2003; Held and Soden, 2006; Sun *et al*., 2007), and the decrease of moderate precipitation events has also been demonstrated in the literature (Fujibe *et al*., 2005; Sun *et al*., 2007; Shiu *et al*., 2012). However, previous studies investigating the precipitation categories are often based on limited bins at constant intervals. By studying the same problem with finer intervals, more details in the precipitation spectral change in the future warmer climate are revealed, especially the imprint of the weakening of the overturning wind on the spectral Centre 1 over all three latitudinal belts, a phenomenon warranting further investigation.

## 4 SUMMARY AND DISCUSSION

Precipitation characteristic changes with global warming are explored using the high‐resolution simulations of ECMWF IFS. The precipitation PDFs are constructed and compared between present‐day and future warming conditions. The result shows a consistent increase at all precipitation intensities at high latitudes (70°–90°), and more frequency occurrence of LP and HP at the expense of MP from the Tropics to midlatitudes under climate warming. The latter pattern of changes can also be characterized as a flatter distribution of precipitation PDFs for a large swath of the globe except the Equator and high latitudes.

As for the precipitation PDF change in different climate zones with global warming, a HP and LP increase accompanied by a MP decrease is displayed in the tropical belt. Over the subtropical and mid‐to‐high latitude land, the PDF change shows a shift towards higher intensity in both summer and winter, but a flatter distribution is found for the subtropical ocean. Summer precipitation over the ocean will acquire a flatter PDF, while the winter ocean will witness a shift to higher intensities in the PDF under warming. In fact, all of the climatological zones except the very dry deserts (*R*_clim_ < 0.1 mm/day) exhibit a common increase in HP and decrease in MP. But LP changes vary with seasons and underlying surfaces. The reduced MP in richer precipitation zones resides in the PDF peak intensities identified as Centre 1, which increase linearly with the precipitation climatology zones. This character is more prominent in the Tropics.

The precipitation PDF districted based on different precipitation climate zones might be the most novel aspect of this study, with intriguing discoveries of two spectral centres. Similar analysis of both observational and modelling datasets is encouraged. The revelation of the ubiquitous reduction of frequency at one of the spectral centres across different climate zones has been linked to the weakening of the circulation; the exact mechanisms should be of interest to the climate‐change dynamics community. And the robustness of this change in precipitation distribution is worthy of verification with other high‐resolution model experiments.
